# Evaluation of the effect of sagging correction calibration errors in radiotherapy software on image matching

**DOI:** 10.1007/s13246-024-01388-y

**Published:** 2024-02-19

**Authors:** Yumi Yamazawa, Akitane Osaka, Yasushi Fujii, Takahiro Nakayama, Kunio Nishioka, Yoshinori Tanabe

**Affiliations:** 1 Department of Radiology, Niigata Prefectural Central Hospital, 205, Shin-minamimachi, Niigata, 205943-0192 Japan; 2 Department of Radiology, Chugoku Central Hospital of the Mutual Aid Association of Public School Teachers, 148-13, Miyuki, Fukuyama, Hiroshima 720-2121 Japan; 3 Department of Radiology, Tokuyama Central Hospital, 1-1 Kodacho, Shunan, Yamaguchi 745-8522 Japan; 4 Faculty of Medicine, Graduate School of Health Sciences, Okayama University, 2-5-1, Shikata, Kita, Okayama, 700-8525 Japan

**Keywords:** Radiotherapy, Sagging correction, Image matching, Winston–Lutz test, Deformable registration

## Abstract

To investigate the impact of sagging correction calibration errors in radiotherapy software on image matching. Three software applications were used, with and without a polymethyl methacrylate rod supporting the ball bearings (BB). The calibration error for sagging correction across nine flex maps (FMs) was determined by shifting the BB positions along the Left–Right (LR), Gun–Target (GT), and Up–Down (UD) directions from the reference point. Lucy and pelvic phantom cone-beam computed tomography (CBCT) images underwent auto-matching after modifying each FM. Image deformation was assessed in orthogonal CBCT planes, and the correlations among BB shift magnitude, deformation vector value, and differences in auto-matching were analyzed. The average difference in analysis results among the three softwares for the Winston–Lutz test was within 0.1 mm. The determination coefficients (R^2^) between the BB shift amount and Lucy phantom matching error in each FM were 0.99, 0.99, and 1.00 in the LR-, GT-, and UD-directions, respectively. The pelvis phantom demonstrated no cross-correlation in the GT direction during auto-matching error evaluation using each FM. The correlation coefficient (r) between the BB shift and the deformation vector value was 0.95 on average for all image planes. Slight differences were observed among software in the evaluation of the Winston–Lutz test. The sagging correction calibration error in the radiotherapy imaging system was caused by an auto-matching error of the phantom and deformation of CBCT images.

## Introduction

Image matching technology is widely used in stereotactic and intensity-modulated radiotherapy to perform high positional accuracy treatment. Quality assurance (QA) of the treatment isocenter and radiographic imaging system is a crucial process that ensures the consistent and accurate alignment of the treatment isocenter and radiographic imaging system [1].

Mechanical, radiation, and imaging isocenters are evaluated for QA [2]. In stereotactic radiotherapy, a central accuracy of 1-mm radius is recommended for the radiation isocenter [1, 2]. The Winston–Lutz (W–L) test and starshot analysis are used in the analysis and adjustment of the radiation isocenter using in-house and vendor software [3, 4].

Ideally, the centers of gravity indicated by the mechanical, radiation, and image isocenters should coincide [5]. However, radiation isocenters differ in radiation focus and beam alignment, leading to slight discrepancies between the radiation and mechanical isocenters of each energy mode [6]. The image isocenter serves as the center of geometric coordinate system for cone-beam computed tomography (CBCT) or 2D X-rays, and the geometric coordinate system is correlated with the mechanical and radiation isocenters through a calibration process [1, 7].

The calibration of geometric coordinates for the image matching system is involved in CBCT image reconstruction, and radiation therapy device manufacturers have implemented some calibration software [8, 9]. The image isocenter is calculated using geometry phantoms with a marker, including a ball-bearing (BB) calibration phantom and a radiation matching system in all directions [7, 10, 11]. In previous studies, vendors’ calibration software reported incorrectly calculated position coordinates up to 0.13 mm in the gun–target (GT) direction due to image analysis error for the W–L test [7]. The geometric coordinates for the image matching system in the Elekta linear accelerator are calibrated in the software using the BB positioned at the radiation isocenter [7]. However, when employing a center-only phantom including a BB for sagging correction, reports regarding the effects on the reconstruction algorithm and images beyond the central region are unavailable. In previous reports of calibration differences in geometric coordinates and misalignment of the radiation isocenter phantom had minimal impact on calibration using cylinder phantom with 16 tungsten-carbide BBs [12].

The development algorithm of the vendor is often a black box; however, the algorithm may be inferred from the results using auto-matching and image analysis as a vector map of deformable image registration (DIR) and temporal subtraction [13, 14]. The DIR is a useful tool for objectively evaluating changes between images using non-rigid registration [13, 14]. Therefore, we considered the possibility of evaluating the geometric coordinate calibration characteristics for the image matching system for a centered-only BB phantom by comparing the results of multiple devices and various calibration results. To the best of our knowledge, there are no reports on the impact of image matching on the sagging correction calibration error of software in radiotherapy using rigid and non-rigid registration. Therefore, we compared the analysis results of radiation isocenters using multiple analysis software programs and assessed the discrepancies in image isocenters and spatial coordinates resulting from these corrections. This study will enable us to evaluate the image matching risk following the correction of radial misalignment discrepancies. Additionally, it will assist us in determining margins for stereotactic radiotherapy and ensure image-guided radiotherapy treatment quality control.

## Methods

### Materials

Three Elekta radiation therapy machines: VersaHD HD (machines A and B) (Elekta AB, Stockholm, Sweden) and Infinity (machine C) (Elekta AB, Stockholm, Sweden) were used in this study. The W–L test was analyzed using three software: Dose LAB (Mobius Medical Systems, Houston, TX, USA), DD system (R-Tech, Tokyo, Japan), and SNC machine (Sun Nuclear Corp., Melbourne, FL). Three kinds of phantoms were used: Lucy quality assurance phantom (Standard Imaging Inc., Middleton, WI, USA), Pelvis phantom, and Geometry phantom.

Flex maps (FMs) were generated using a perfectly spherical metal ball and a dial gauge with 0.01 mm precision as adjustments were made. The positions of the BB were fine-tuned for the radiating isocenter of the linac through analysis of the W–L test at 12 different angles and collimators. This was achieved using Elekta XVI software version R7: the latest version (machine A, machine C), version R6: the previous version (machine B). The alignment of the kilovoltage (kV) imaging and megavoltage (MV) treatment isocenters was corrected using BB imaging of the analyzed position. In this study, we evaluated the image matching effect following the sagging correction calibration errors through the analysis of radiation and image isocenters. This evaluation was performed using the Elekta XVI software and the W–L test.

### W–L test analysis with and without the polymethyl methacrylate rod using multiple analysis software

**Fig. 1:**
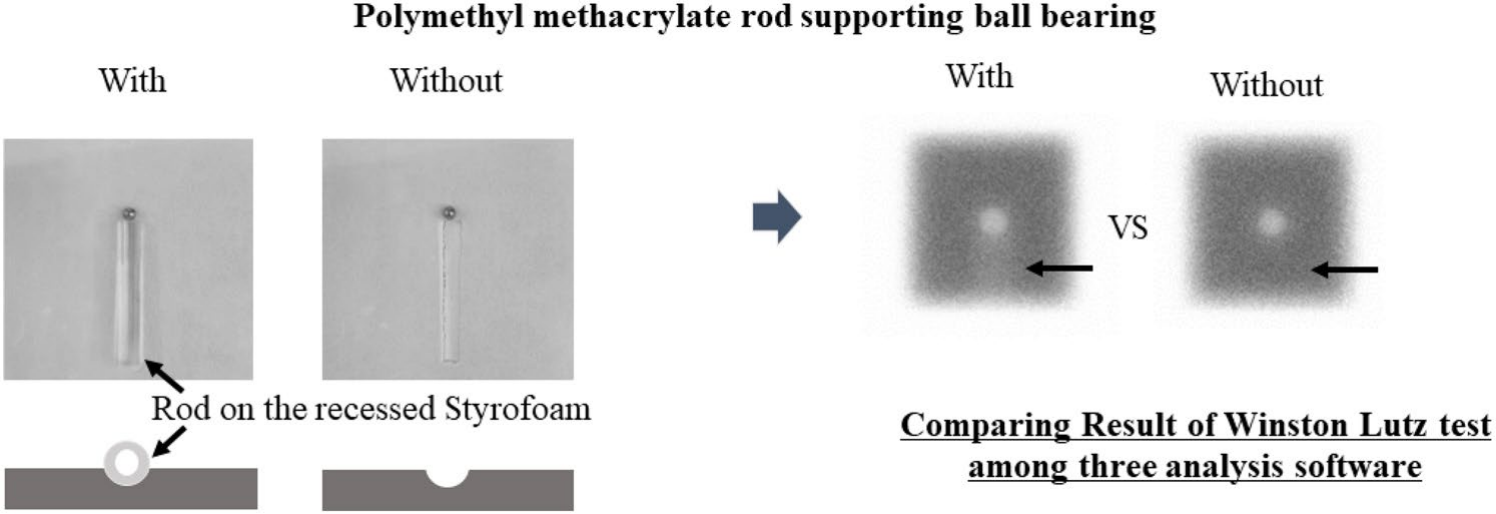
Analysis of the Winston Lutz test with and without the polymethyl methacrylate rod supporting the BB

The influence of the presence or absence of a polymethyl methacrylate rod supporting the BB was evaluated among three analytical software (Fig. 1). The Styrofoam was recessed so that the polymethyl methacrylate rod can be easily removed to prevent the iron ball from shifting (Fig. 1). The W–L test was performed with and without the polymethyl methacrylate rod supporting BB using Elekta XVI software. This W–L test was conducted on a table couch at a 0° gantry, 0° collimator, and an irradiation field diameter of 2–10 cm^2^ (1.0 cm^2^ step). The results of the W–L test with and without the imprint of the polymethyl methacrylate rod support were compared among three different analysis software.

The Styrofoam was placed on the table couch in a manner that allowed the polymethyl methacrylate rod to be easily placed on and removed from the recessed Styrofoam.

### Acquisition of calibration data of 10 different FMs by changing the BB

**Fig. 2:**
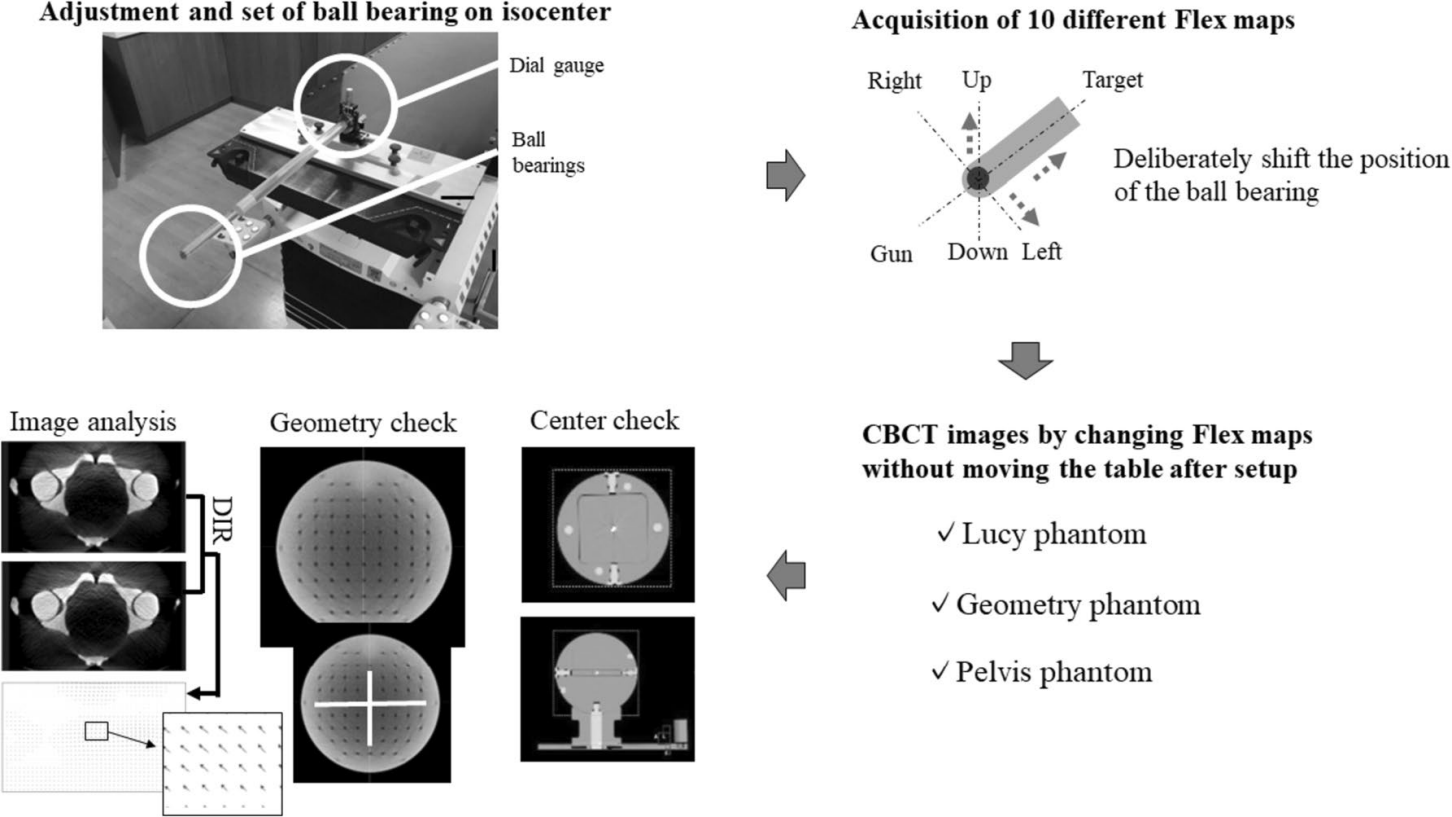
The flowchart of this study for 10 different flex maps

The W–L tests were performed on the BB at 12 different angles and collimator settings and were analyzed using Elekta XVI software. The reference FM was obtained by positioning the BB within 0.05 mm in all directions. The position of the BB at the reference was shifted by 0.2, 0.4, and 0.6 mm in the Left–Right (LR), GT, and Up–Down (UD) directions using a dial gauge. Subsequently, nine FMs with the sagging correction calibration error were obtained. Figure 2 presents an assessment of 10 different FMs, along with a flowchart illustrating the impact of sagging correction calibration errors on image matching using these 10 FMs.

### Auto-matching evaluation of phantoms by changing 10 different FMs

The Lucy quality assurance phantom with the tungsten sphere in the center and pelvic phantom were scanned using a CT system. Subsequently, a registration image was created using the Monaco radiation therapy planning system (Elekta AB, Stockholm, Sweden). The Lucy phantom was positioned at the isocenter, and the CBCT was performed by changing 10 types of FMs without moving the position of the Lucy phantom. The CBCTs were scanned in the half-fan mode, with 100 kVp, 20 mA/frame, 10 ms/frame, for a total of 183 frames. Subsequently, the registration error of auto-matching the scanned CBCT image with the planning CT by the radiation therapy planning system was calculated. The relationship between the BB shifts (x, y) of the FMs and the registration error ($\overline{x },\overline{y })$ in auto-matching was determined using the following formula [15]:

1 $$Cross~correlation~coefficient\left( {X,Y} \right) = \frac{{\sum (x - \bar{x})\sum \left( {y - \bar{y}~} \right)}}{{\sqrt {\sum (x - \bar{x})^{2} \sum (y - \bar{y})^{2} } }}$$

Next, the cross-correlation coefficients between the auto-matching registration error and the BB shift amounts of the FMs of the pelvic phantom were calculated using the same formula.

The cross-correlation coefficients between the BB shift amount of each FM and the auto-matching registration error of auto-matching were calculated. The CBCTs images of the pelvic phantom were scanned in the half-fan mode, with 120 kVp, 80 mA/frame, and 40 ms/frame, for a total of 183 frames.

### Evaluation of geometric information change beyond the center using geometry phantom

The geometry phantom has markers at 20-mm intervals, as shown in Fig. 2, and the geometric position can be evaluated. In the two VersaHD, the CBCT images of the geometry phantom were scanned by changing 10 types of FMs. The axial plane of the CBCT images of the geometry phantom was used to evaluate the profile of each FM in the LR direction (− 105 to 105 mm) and GT direction (− 65 to 65 mm) using ImageJ/Fiji version 1.53f51 (National Institutes of Health, Bethesda, MD, USA). Thereafter, the distance between two markers (LR direction: − 20 to 20, − 40 to 40, − 60 to 60, − 80 to 80, and − 100 to 100 mm; GT direction: − 20 to 20, − 40 to 40, − 60 to 60) was calculated for each FM. The spatial coordinate differences of the markers with and without the BB shift amounts were calculated for each marker, and the differences in the spatial coordinates for each FM were averaged.

### Evaluation between images with modified FMs using deformable image registration

Three orthogonal planes (axial, coronal, and sagittal) for the pelvic CBCT images were created for each FM. The bUnwarpJ function of ImageJ/Fiji was used to perform DIR between the image of the reference FM and calibration error images of the nine different FMs. The DIR uses the signal values of the reference image (Id(x)) and the corresponding signal values (Ii(g(x))) from the generated images by the displaced FM as features. It uses an approximation based on the B-spline function to calculate the distance between these features, resulting in a deformation vector. The optimal direct consistency error as a deformation vector was determined using the following formula, computed as quantities [16]:

2 $$Deformation~vector~value = \sum {\left[ {Id\left( x \right) - Ii\left( {g\left( x \right)} \right)} \right]^{2} }$$

The correlation between the BB shift amount and the deformation vector value of each FM was analyzed by a linear approximation using the statistical software SPSS.

## Results

Table 1 shown the W–L test results for each irradiation field size, comparing cases with and without the polymethyl methacrylate rod supporting the BB. The analysis differences of the W–L test results among the SNC, DD system, and Dose LAB software was 0.00 mm median (standard deviation (SD): 0.1 mm, range: − 0.26 to 0.29 mm). In the evaluation for with and without the polymethyl methacrylate rod supporting the BB, the median difference (range) in the overall LR and GT directions for the SNC, DD system, and Dose LAB software, were -0.03 mm (− 0.14 to 0.11 mm), 0.00 mm (− 0.10 to 0.29 mm), and − 0.03 mm (− 0.13 to 0.11), respectively. The SD of the W–L test results between each irradiation field size were 0.07 mm, 0.05 mm, and 0.05 mm for SNC, DD system, and Dose LAB, respectively.

**Table 1:** Results of the Winston–Lutz test of different irradiation field sizes with and without the polymethyl methacrylate rod supporting the ball bearing

	Analysis software	Direction	Field size (cm^2^)									
			2	3	4	5	6	7	8	9	10	Average
Rod (−)	SNC	LR	− 0.27	− 0.17	− 0.18	− 0.12	− 0.2	− 0.23	− 0.2	− 0.21	− 0.24	− 0.20
		GT	0.73	0.69	0.85	0.79	0.86	0.77	0.71	0.7	0.79	0.77
		Total	0.78	0.71	0.87	0.8	0.88	0.81	0.74	0.73	0.82	0.79
	D–D	LR	− 0.22	− 0.14	− 0.16	− 0.06	− 0.15	− 0.22	− 0.21	− 0.21	− 0.19	− 0.17
		GT	0.79	0.75	0.88	0.86	0.86	0.82	0.82	0.72	0.79	0.81
		Total	0.82	0.76	0.89	0.86	0.87	0.85	0.84	0.75	0.82	0.83
	Dose LAB	LR	− 0.25	− 0.16	− 0.18	− 0.13	− 0.20	− 0.23	− 0.21	− 0.22	− 0.17	− 0.19
		GT	0.77	0.75	0.88	0.83	0.82	0.77	0.76	0.74	0.77	0.79
		Total	0.81	0.77	0.90	0.84	0.84	0.80	0.79	0.77	0.79	0.81
Rod (+)	SNC	LR	− 0.18	− 0.26	− 0.1	− 0.13	− 0.3	− 0.23	− 0.2	− 0.2	− 0.37	− 0.22
		GT	0.79	0.73	0.88	0.84	0.88	0.9	0.76	0.73	0.84	0.82
		Total	0.81	0.77	0.89	0.85	0.93	0.93	0.78	0.76	0.91	0.85
	D–D	LR	− 0.16	− 0.20	− 0.13	− 0.13	− 0.29	− 0.16	− 0.21	− 0.21	− 0.39	− 0.21
		GT	0.82	0.81	0.88	0.80	0.84	0.86	0.80	0.73	0.88	0.82
		Total	0.84	0.84	0.89	0.81	0.88	0.87	0.83	0.76	0.96	0.85
	Dose LAB	LR	− 0.39	− 0.13	− 0.19	− 0.41	− 0.20	− 0.18	− 0.13	− 0.13	− 0.13	− 0.21
		GT	0.86	0.86	0.77	0.84	0.79	0.80	0.76	0.82	0.79	0.81
		Total	0.94	0.87	0.79	0.93	0.81	0.82	0.77	0.83	0.80	0.84

**Fig. 3:**
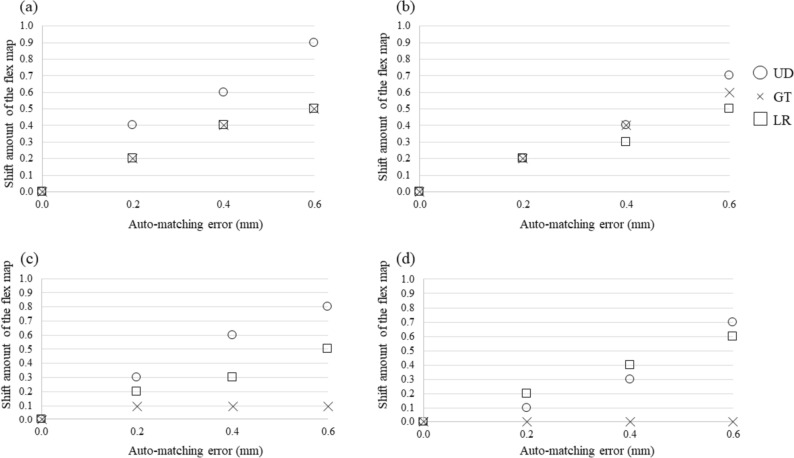
The relationship between the BB shift amount and the deformation vector value of each FM **a** Lucy phantom, machine with latest version software radiotherapy machine. **b** Lucy phantom, machine with previous version software radiotherapy machine. **c** Pelvis phantom, machine with latest version software radiotherapy machine. **d** Pelvis phantom, machine with previous version software radiotherapy machine

In the auto-matching evaluation, registration errors occurred in the Lucy phantom according to the BB shift amount of the FMs, whereas there was an irregular registration error occurred for the BB shift amount of the FMs in the GT direction in the pelvis phantom (Table 2). The cross-correlation coefficients between the BB shift amount and registration error of the auto-matching in the Lucy phantom in the LR, GT, and UD directions were 0.992, 1.000, and 0.990, respectively, in the latest version software radiotherapy machine, and 0.994, 1.000, and 0.992, respectively, in the previous version software radiotherapy machine (Fig. 3). Similarly, the cross-correlation coefficients of the pelvic phantom in the LR, GT, and UD directions were 0.996, 0.775, and 0.992, respectively, in the latest version software radiotherapy machine, and 1.000, no cross-correlation, and 0.959, respectively, in the previous version software radiotherapy machine (Fig. 3).

**Table 2:** Relationship between flex map and phantom auto-matching with added ball bearing shift

Shift amount of BB for flex map		Shift	LR (mm)				GT (mm)				UD (mm)			
			0.0	0.2	0.4	0.6	0.0	0.2	0.4	0.6	0.0	0.2	0.4	0.6
Lucy	A	LR (mm)	0.0	0.4	0.6	0.9	0.0	0.1	0.1	0.0	0.0	0.1	0.0	0.1
		GT (mm)	0.0	0.0	0.0	0.0	0.0	0.2	0.4	0.6	0.0	0.0	0.0	0.0
		UD (mm)	0.0	0.0	0.0	0.0	0.0	0.0	0.0	0.0	0.0	0.2	0.4	0.5
	B	LR (mm)	0.0	0.2	0.4	0.7	0.0	0.1	0.1	0.1	0.0	0.0	0.0	0.0
		GT (mm)	0.0	0.0	0.0	0.0	0.0	0.2	0.4	0.6	0.0	0.0	0.0	0.0
		UD (mm)	0.0	0.0	0.0	0.0	0.0	0.0	0.0	0.0	0.0	0.2	0.3	0.5
	C	LR (mm)	0.0	0.2	0.3	0.6	0.0	0.0	0.0	0.0	0.0	0.0	0.0	0.0
		GT (mm)	0.0	0.0	0.0	0.0	0.0	0.2	0.4	0.6	0.0	0.0	0.0	0.0
		UD (mm)	0.0	0.0	0.0	0.0	0.0	0.0	0.0	0.0	0.0	0.2	0.4	0.6
Pelvis	A	LR (mm)	0.0	0.3	0.8	0.9	0.0	0.0	0.1	0.0	0.0	0.1	0.1	0.0
		GT (mm)	0.0	0.0	0.1	0.0	0.0	0.1	0.1	0.7	0.0	0.0	0.1	0.1
		UD (mm)	0.0	0.0	0.0	0.0	0.0	0.0	0.1	0.1	0.0	0.1	0.3	0.5
	B	LR (mm)	0.0	0.6	0.6	0.8	0.0	0.0	0.0	0.0	0.0	0.0	0.0	0.0
		GT (mm)	0.0	0.2	0.1	0.0	0.0	0.1	0.3	0.6	0.0	0.0	0.0	0.0
		UD (mm)	0.0	0.1	0.0	0.0	0.0	0.0	0.0	0.0	0.0	0.2	0.4	0.5

Table 3 show the results of the geometry phantom evaluation. The profile in the LR direction shifted the spatial coordinates of the markers in only the LR-shifted FMs (Table 3). Similarly, the profile in the GT direction shifted the spatial coordinates of the markers in only the GT-shifted FMs (Table 3). The distance between two markers was equivalent to the slight difference between each FM; the error in the distance increased with the distance from the center (40 mm: 0.69 mm, 80 mm: 1.02 mm, 120 mm: 1.40 mm).

**Table 3:** Results of the geometry phantom

Shift amount of BB for flex map (mm)			A				B			
			0	0.2	0.4	0.6	0	0.2	0.4	0.6
LR profile (image with Flex map for LR direction shift)	The difference of spiral coordinate of marker (mm)	Ave.	_	− 0.26	− 0.45	− 0.51	_	− 0.11	− 0.44	− 0.63
		SD	_	0.19	0.31	0.60	_	0.20	0.22	0.25
	The distance between two markers (mm)	40	39.3	39.2	38.9	37.8	39.9	39.5	39.3	39.6
		80	79.1	78.8	78.9	78.9	78.6	78.9	78.8	78.9
		120	119.0	119.0	118.7	118.4	118.1	118.4	118.2	118.4
		160	158.7	158.7	158.1	158.3	157.8	157.8	157.7	157.7
		200	203.4	203.0	203.1	202.5	202.1	201.8	201.8	201.8
GT profile (image with Flex map for GT direction shift)	The difference of spiral coordinate of marker (mm)	Ave.	_	0.00	0.11	0.34	_	0.19	0.24	0.45
		SD	_	0.13	0.21	0.19	_	0.31	0.21	0.17
	The distance between two markers (mm)	40	39.3	39.2	39.5	39.3	39.3	39.9	39.6	39.6
		80	79.1	79.1	79.2	79.7	78.9	78.9	79.1	79.2
		120	119.0	118.8	118.5	119.0	118.8	118.7	118.5	118.7

**Fig. 4:**
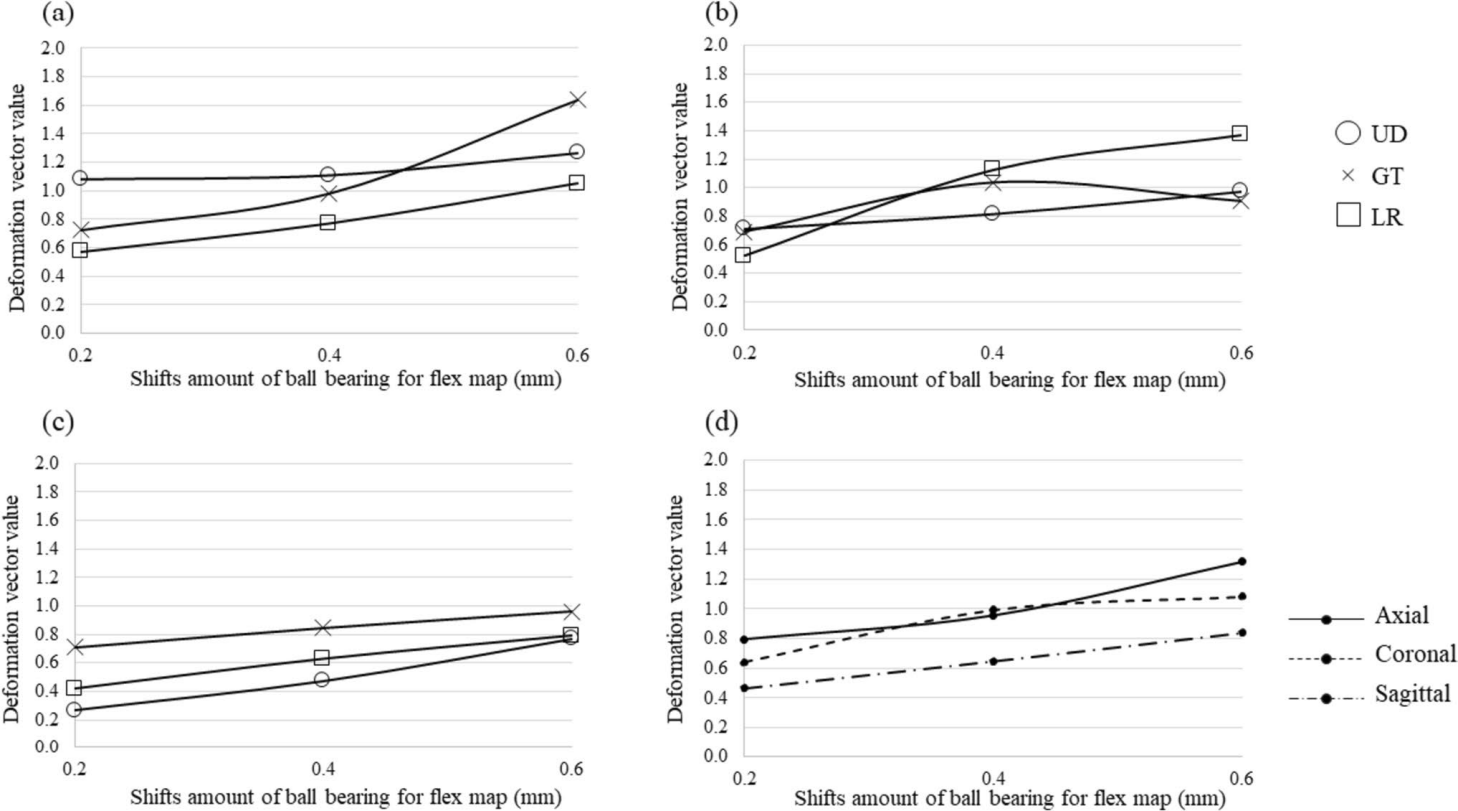
The relationship between the BB shift of the FMs and deformation vector value **a** axial, **b** coronal, **c** sagittal, and **d** total averages

As shown in Fig. 4, the deformation vector value of the pelvic phantom was increased according to the BB shift amount in the axial and sagittal plane images. There was a slight decrease in the deformation vector value from 0.4–0.6 mm in the GT direction of the coronal plane image (Fig. 4b). The correlation coefficients (r) between the BB shift amount and the average deformation vector value in the three orthogonal planes (axial, coronal, and sagittal) in the LR, GT, and UD directions were 0.95, 0.90, and 1.00, respectively.

## Discussion

This study evaluated the image matching effect by creating sagging correction calibration errors through the analysis process of the radiation and image isocenters. Evaluation of the effect of the polymethyl methacrylate rod support BB on the image analysis processing using W–L test analysis revealed small differences (SD, 0.1 mm; maximum difference, 0.29 mm) between the software. Understanding the uncertainties and systematic errors of the software in high accuracy stereotactic radiotherapy is crucial. During the W–L test analysis, of each software, which involved changing the irradiation field with and without the polymethyl methacrylate rod supporting the BB, revealed a maximum difference of 0.29 mm was observed in the rod direction. Although the polymethyl methacrylate rod supporting the BB may affect the analysis results, we believe that other complex factors, such as jaw positioning accuracy, may also be involved [17].

This study created FMs with calibration errors by shifting positioning set BBs, assuming analysis errors in registration, to evaluate the influence of software, multileaf collimator, jaw, beam alignment, and other radiographic image matching systems on sagging correction. In the evaluation of the Lucy phantom with the tungsten sphere in the center, the auto-matching error was correlated with the BB shift amount of the FMs. The spatial coordinate of the marker for the geometry phantom was also shifted according to the BB shift amount of the FMs. In this study, the results of the Lucy phantom suggest that errors in sagging correction directly affect the center coordinates and indicate the importance of analysis and placement for BB placement during FM acquisition.

The correlation result of auto-matching for the pelvic phantom was low compared with that of the Lucy phantom, and was the difference between each radiotherapy machine. Additionally, the error in the distance between the geometry phantom markers increased with the distance from the center. As this method of sagging correction utilizing only the BB provides information primarily regarding the center, it may not offer sufficient correction for the non-center areas [12].

Auto-matching results for the pelvic phantom demonstrated no movement equivalent to the FM shift in the GT direction. However, there was a strong correlation between the BB shift amount of the FMs and the deformation vector value using DIR in the pelvic phantom. These results suggest that the FM shift during the sagging correction may cause a slight deformation of the entire image during image generation, excluding the center. Additionally, the difference in auto-matching results between devices may arise from image generation using a predefined manufacturer correction algorithm, rather than correcting for the sagging of each device in off-center spatial coordinates.

This study had some limitations. First, there is uncertainty in the evaluation results due to the use of only two devices. However, both devices exhibited similar characteristics; registration errors during sagging correction of the radiographic image matching system affected the image matching results. Second, the evaluation of image deformation was conducted as an overall evaluation without considering the influence of detailed spatial coordinates. Nonetheless, these limitations highlight the risks associated with sagging correction in radiation therapy apparatus and assist in considering the risks of reducing the treatment plan margin [18]. In the future, we believe that evaluating known phantoms for established spatial coordinates will provide further clarification.

## Conclusion

Slight differences were observed among software in the W–L test analysis, which evaluates radiation isocenters. This study suggests that the FM shift during radiotherapy equipment registration, intended for correction of radiotherapy sagging, caused systematic errors in the phantom images. Caution is required when performing sagging correction calibration in the radiotherapy imaging system, as it directly affects the accuracy of equipment alignment and the shift of spatial coordinates beyond the image center.

## Data Availability

The data used to support the findings of this study are available from the corresponding authors upon request.
